# Study of Functional Outcomes of the Ilizarov External Fixator in the Treatment of Open Tibial Fractures (Gustilo-Anderson Grades II, IIIA, and IIIB): A Prospective Clinical Study

**DOI:** 10.7759/cureus.107018

**Published:** 2026-04-14

**Authors:** Midhun Romeo, Ashok Nayak, Srikant Kulkarni

**Affiliations:** 1 Orthopaedics, Bijapur Lingayat District Educational Association (BLDE) (Deemed to be University), Shri B M Patil Medical College, Hospital and Research Centre, Vijayapura, IND

**Keywords:** external fixation, fracture union, functional outcome, ilizarov fixator, open tibia fracture

## Abstract

Introduction: Open fractures of the tibia are commonly associated with significant soft tissue injury and high risk of complications, posing a challenge in achieving optimal functional outcomes. The Ilizarov external fixator offers stable fixation with minimal soft tissue disruption and allows early mobilization. The present study aimed to evaluate the functional outcomes of the Ilizarov external fixator in the management of open tibial fractures (Gustilo-Anderson grades II, IIIA, and IIIB).

Materials and methods: This prospective observational study was conducted in the Department of Orthopaedics at Shri B. M. Patil Medical College, Hospital and Research Centre, Vijayapura, Karnataka. A total of 20 patients aged 18-65 years with open tibial fractures were included. All patients underwent initial surgical debridement, following which fractures were classified according to the Gustilo-Anderson classification and subsequently managed with Ilizarov external fixation. Clinical, radiological, and functional parameters were assessed during follow-up. Functional outcome was evaluated using the Johner and Wruhs criteria, a fracture-specific scoring system incorporating clinical, radiological, and functional parameters.

Results: Among 20 patients, the majority were males (17 (85.0%)) and in the age group of 20-40 years (15 (75.0%)). Road traffic accidents were the most common mode of injury (15 (75.0%)). Grade IIIB fractures were predominant (10 (50.0%)), and distal third fractures were most common (12 (60.0%)). Early fixation within 24 hours was achieved in 17 (85.0%) patients. Bone loss was present in seven (35.0%) patients, and corticotomy was performed in six (30.0%). Pin tract infection occurred in 11 (55.0%) patients, predominantly grade II (8 (40.0%)), with no deep infections (0 (0.0%)). All patients achieved fracture union (20 (100.0%)) with satisfactory functional outcomes; excellent to good results were observed in 18 (90.0%) patients. Early weight bearing was achieved in all patients (20 (100.0%)).

Conclusion: The Ilizarov external fixator is an effective modality for the management of open tibial fractures, providing stable fixation, early mobilization, and satisfactory functional outcomes. However, findings should be interpreted cautiously, and further large-scale studies are required to validate these results.

## Introduction

Open fractures of the tibia represent a significant proportion of orthopaedic trauma due to the subcutaneous location of the bone and its vulnerability to high-energy injuries [1]. These fractures are frequently associated with extensive soft tissue damage, contamination, and a higher risk of complications such as infection, delayed union, and non-union [2]. The Gustilo-Anderson classification remains the most widely used system for categorizing open fractures and provides important prognostic information based on the severity of soft tissue injury and contamination, and is typically assigned after initial surgical debridement to allow accurate assessment of wound characteristics [3].

The management of open tibial fractures has evolved from conservative approaches to early surgical intervention emphasizing thorough debridement, stable fixation, and timely soft tissue coverage [1,4]. While intramedullary nailing is commonly used in selected cases, external fixation continues to play a crucial role, particularly in high-grade open fractures with significant soft tissue compromise [5]. The choice of fixation method is influenced by fracture pattern, degree of contamination, and overall patient condition [6].

The Ilizarov external fixator, based on the principle of tension-stress, offers several advantages in managing complex open tibial fractures [7]. It provides stable fixation with minimal additional soft tissue disruption, allows early weight bearing, and enables correction of deformities and management of bone loss [8]. Furthermore, it facilitates repeated wound care procedures without compromising fracture stability [8]. Despite these advantages, complications such as pin tract infection and joint stiffness may occur, necessitating careful evaluation of outcomes [9].

Although previous studies have reported favourable outcomes with the Ilizarov technique, variability in patient profiles, fracture severity, and outcome measures necessitates further prospective evaluation [10,11]. Additionally, differences in functional outcome assessment tools across studies highlight the need for use of standardized and fracture-specific scoring systems to allow meaningful comparison of results. In this context, the present study was aimed to assess the functional outcome of Ilizarov external fixator in the treatment of open tibial fractures (Gustilo-Anderson grades II, IIIA, and IIIB), with emphasis on fracture union, time to union, functional parameters, complications, and patient satisfaction.

This research work was originally conducted as part of a postgraduate dissertation submitted to the Department of Orthopaedics, Bijapur Lingayat District Educational Association (BLDE) (Deemed to be University), Shri B M Patil Medical College, Hospital and Research Centre, Vijayapura, Karnataka, India.

## Materials and methods

This prospective observational clinical study was conducted in the Department of Orthopaedics at Bijapur Lingayat District Educational Association (BLDE) (Deemed to be University), Shri B M Patil Medical College, Hospital and Research Centre, Vijayapura, India, from June 2024 to December 2025, after obtaining approval from the Institutional Ethics Committee (IEC No: BLDE(DU)/IEC-SBMPMC/141/2023-24). Written informed consent was obtained from all patients prior to inclusion.

The sample size was calculated using the single proportion formula based on a previously reported union rate of 74.5% by Thomas and Nishara [12]. With a confidence level of 95% (Z = 1.96) and an allowable error of 20%, the sample size was calculated as:

\[ n = \frac{Z^2 \cdot p \cdot q}{d^2} \]

The calculated sample size was 18, which was rounded off to 20 patients for the present study. A total of 20 consecutive patients aged 18-65 years with open tibial fractures classified as Gustilo-Anderson grades II, IIIA, and IIIB were included. Patients presenting within 24 hours of injury and willing to undergo follow-up were enrolled. Patients with Gustilo-Anderson grades I and IIIC fractures, pathological fractures, vascular injury requiring repair, ipsilateral femoral fractures, polytrauma affecting mobilization, pre-existing deformities, active infection, metabolic bone disease, or those unwilling to undergo follow-up were excluded.

All patients were initially managed according to hospital protocols. Clinical assessment included documentation of age, sex, side involved, mode of injury, and time to presentation. Fractures were classified using the Gustilo-Anderson classification after initial surgical debridement to allow accurate assessment of soft tissue injury and contamination, and anatomical location of fracture (proximal, middle, distal third) was noted [13]. Radiological evaluation included standard anteroposterior and lateral radiographs of the tibia including knee and ankle joints.

All patients underwent early surgical debridement followed by application of the Ilizarov circular external fixator under spinal or general anaesthesia [14]. The timing of fixator application, presence of bone loss, need for corticotomy, and type of wound management (delayed primary closure, secondary intention, skin grafting, or flap coverage) were recorded. Bone loss, where present, was measured in centimetres.

Postoperatively, patients received antibiotics, analgesia, and standardized pin site care. Early mobilization and physiotherapy were initiated. Weight bearing status, including time to initiation and progression to full weight bearing, was documented. Patients were followed up at regular intervals (at two weeks, six weeks, and monthly thereafter until union, followed by periodic assessments) until fracture union and thereafter. Outcome assessment included evaluation of complications such as pin tract infection, pain at fracture site, and deep infection [15]. Radiological and functional parameters assessed included knee flexion, knee extension lag, ankle range of motion, limb length discrepancy, and angular deformity in coronal and sagittal planes.

**Figure 1:**
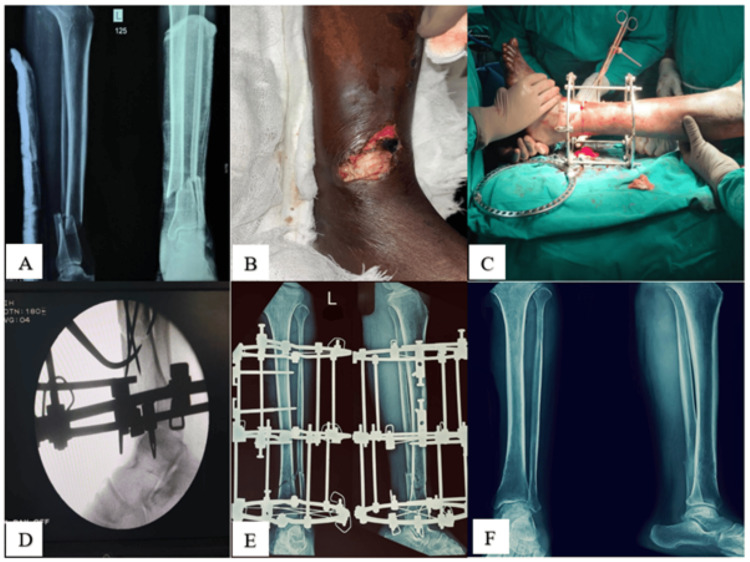
Representative Case of Gustilo-Anderson Grade IIIB Open Tibial Fracture Managed With Ilizarov External Fixator (A) Preoperative radiograph showing tibial fracture; (B) preoperative clinical photograph demonstrating soft tissue defect; (C) intraoperative image during application of Ilizarov external fixator; (D) intraoperative C-arm image confirming fracture alignment; (E) postoperative radiograph showing fixator in situ; and (F) follow-up radiograph demonstrating fracture union.

Fracture union was defined as the presence of bridging callus in at least three cortices on radiographs along with absence of pain at the fracture site during weight bearing. Fracture union was assessed clinically and radiologically. Time to union (in weeks), hospital stay duration, and time to return to work were recorded. Pain was assessed using the Visual Analog Scale (VAS) [16]. Functional outcome was evaluated using the Johner and Wruhs criteria and categorized as excellent, good, fair, or poor [17]. The Johner and Wruhs criteria were chosen as they provide a comprehensive fracture-specific assessment incorporating clinical, radiological, and functional parameters, facilitating comparison with previous Ilizarov studies (Figure 1).

Statistical analysis was performed using appropriate software (IBM SPSS Statistics version 26, IBM Corp., Armonk, NY). Continuous variables were expressed as mean ± standard deviation, and categorical variables as frequencies and percentages and analysed using Fisher’s Exact test. A p-value of <0.05 was considered statistically significant.

## Results

The study population predominantly comprised younger individuals aged 20-40 years (15 (75.0%)) with a mean age of 38.4 ± 6.8 years. Males constituted the majority (17 (85.0%)), and the left side was more commonly affected (13 (65.0%)) (Table 1).

**Table 1:** Demographic Characteristics (n = 20) Data are presented as frequency and percentage (n (%)) for categorical variables and mean ± standard deviation (SD) for continuous variables. Descriptive statistics were used to summarize demographic characteristics. No inferential statistical test was applied for this table.

Variable	Category	n (%) / Mean ± SD
Age (years)	20-40	15 (75.0%)
	41-60	5 (25.0%)
	Mean ± SD	38.4 ± 6.8
Sex	Male	17 (85.0%)
	Female	3 (15.0%)
Side affected	Left	13 (65.0%)
	Right	7 (35.0%)

Road traffic accidents were the leading cause of injury (15 (75.0%)). Most patients presented within 24 hours (9 (45.0%)). Grade IIIB fractures were most common (10 (50.0%)), and the distal third of the tibia was the predominant fracture site (12 (60.0%)) (Table 2).

**Table 2:** Injury Profile (n = 20) Data are presented as frequency and percentage (n (%)) for all categorical variables describing the injury profile. Descriptive statistics were used.

Variable	Category	n (%)
Mode of injury	Road traffic accident	15 (75.0%)
	Fall from height	5 (25.0%)
Time to presentation	<24 hours	9 (45.0%)
	24-48 hours	6 (30.0%)
	48-72 hours	5 (25.0%)
Gustilo-Anderson grade	Grade II	6 (30.0%)
	Grade IIIA	4 (20.0%)
	Grade IIIB	10 (50.0%)
Fracture site	Distal third	12 (60.0%)
	Proximal third	7 (35.0%)
	Midshaft	1 (5.0%)

Early application of the Ilizarov fixator was achieved in most patients within one day (17 (85.0%)). Bone loss was observed in seven (35.0%) cases, with a mean bone loss of 3.5 ± 0.9 cm, and corticotomy was performed in six (30.0%). Delayed primary closure (9 (45.0%)) and secondary intention healing (8 (40.0%)) were the most common wound management methods (Table 3).

**Table 3:** Treatment Details (n = 20) Data are presented as frequency and percentage (n (%)) for categorical variables and mean ± standard deviation (SD) for continuous variables related to treatment characteristics. Descriptive statistical analysis was performed.

Variable	Category	n (%) / Mean ± SD
Time to Ilizarov application	Day 1	17 (85.0%)
	Day 2	1 (5.0%)
	Day 3	2 (10.0%)
	Mean ± SD (days)	1.3 ± 0.7
Hospital stay	Mean ± SD (days)	8.3 ± 1.0 days
Bone loss	Yes	7 (35.0%)
	Mean (cm)	3.5 ± 0.9
Corticotomy	Yes	6 (30.0%)
Wound management	Delayed primary closure	9 (45.0%)
	Secondary intention	8 (40.0%)
	Skin graft	2 (10.0%)
	Flap	1 (5.0%)

Pin tract infection was noted in 11 (55.0%) patients, predominantly grade II (8 (40.0%)), while no cases of grade IV infection were observed. Pain at the fracture site was present in six (30.0%) patients, and no deep infections were reported (0 (0.0%)) (Table 4).

**Table 4:** Complications (n = 20) Data are presented as frequency and percentage (n (%)) for categorical variables related to complications. Descriptive statistics were used.

Variable	Category	n (%)
Pin tract infection	None	9 (45.0%)
	Grade II	8 (40.0%)
	Grade III	3 (15.0%)
	Grade IV	0 (0.0%)
Pain at fracture site	Present	6 (30.0%)
Deep infection	Present	0 (0.0%)

Functional outcomes demonstrated good joint mobility, with mean knee flexion of 132.8 ± 7.4° and ankle range of motion of 91.0 ± 7.0% of normal. Residual deformity and limb length discrepancy were minimal, with mean limb length discrepancy of 0.49 ± 0.32 cm and angular deformity within acceptable limits (Table 5).

**Table 5:** Functional and Radiological Parameters (n = 20) Data are presented as mean ± standard deviation (SD) along with range for continuous functional and radiological parameters. Descriptive statistical methods were applied. ROM: range of motion.

Parameter	Mean ± SD	Range (Minimum-Maximum)
Knee flexion (°)	132.8 ± 7.4	115-140
Knee extension lag (°)	7.0 ± 5.8	0-15
Ankle ROM (% of normal)	91.0 ± 7.0	75-100
Limb length discrepancy (cm)	0.49 ± 0.32	0-1.3
Coronal angulation (°)	4.5 ± 3.6	0-12
Sagittal angulation (°)	5.1 ± 2.6	0-10

All patients achieved fracture union (20 (100.0%)) with a mean union time of 24.1 ± 3.4 weeks (range: 19-32 weeks). Early mobilization was evident as all patients initiated weight bearing by day 2 (20 (100.0%)) and achieved full weight bearing at a mean of 1.4 ± 0.5 weeks. The mean time to return to work was 28.5 ± 3.8 weeks (Table 6).

**Table 6:** Healing Parameters (n = 20) Data are presented as frequency and percentage (n (%)) for categorical variables and mean ± standard deviation (SD) with range for continuous healing parameters. Descriptive statistical analysis was performed.

Variable	Value
Union achieved	20 (100%)
Time to union (weeks)	24.1 ± 3.4 (19-32)
Weight bearing initiation	Day 2 (100%)
Full weight bearing	1.4 ± 0.5 weeks
Return to work	28.5 ± 3.8 weeks

Pain at final follow-up was minimal with a mean VAS score of 0.45 ± 1.0. Most patients reported excellent (12 (60.0%)) or good (6 (30.0%)) satisfaction. Overall, excellent outcomes were observed in 15 (75.0%) patients, with no poor outcomes reported (Table 7).

**Table 7:** Patient Assessment (n = 20) Data are presented as frequency and percentage (n (%)) for categorical variables and mean ± standard deviation (SD) for continuous variables assessing patient-reported outcomes. Descriptive statistics were used. VAS: Visual Analog Scale.

Variable	Category	n (%) / Mean ± SD
VAS pain score	Mean ± SD	0.45 ± 1.0
Satisfaction	Excellent	12 (60.0%)
	Good	6 (30.0%)
	Fair	2 (10.0%)
Final outcome	Excellent	15 (75.0%)
	Good	3 (15.0%)
	Fair	2 (10.0%)
	Poor	0

No statistically significant association was found between mode of injury (χ² = 0.799, p = 0.670) or Gustilo-Anderson grade (χ² = 2.533, p = 0.639) and final outcome, indicating consistent effectiveness of the Ilizarov fixator across different injury patterns. Given the small sample size, Fisher’s Exact test was applied where appropriate to ensure validity of results (Table 8).

**Table 8:** Association of Injury Characteristics With Final Outcome (n = 20) Data are presented as frequency and percentage (n (%)). Inferential analysis was performed using the Chi-square test to assess associations between categorical variables; however, in instances where expected cell counts were small, Fisher’s Exact test was applied. A p-value of <0.05 was considered statistically significant. RTA: road traffic accident.

Variable	Category	Excellent n (%)	Good n (%)	Fair n (%)	p-value
Mode of injury	Fall from height	4 (26.7%)	1 (33.3%)	0 (0.0%)	0.670
	RTA	11 (73.3%)	2 (66.7%)	2 (100.0%)	
Gustilo-Anderson grade	Grade II	5 (33.3%)	1 (33.3%)	0 (0.0%)	0.639
	Grade IIIA	3 (20.0%)	1 (33.3%)	0 (0.0%)	
	Grade IIIB	7 (46.7%)	1 (33.3%)	2 (100.0%)	

## Discussion

The demographic profile in the present study showed a predominance of younger individuals with a mean age of 38.4 ± 6.8 years, with 15 (75.0%) patients in the 20-40 years age group and male predominance of 17 (85.0%). These findings are comparable to those of Çelik et al. and Anjum et al., who reported mean ages of 38.2 years and 37.4 years, respectively [18,19]. Road traffic accidents accounted for 15 (75.0%) cases, similar to the findings by Thomas and Nishara and Bhosale and Naikwade [12,20]. The higher proportion of grade IIIB fractures in the present study (10 (50.0%)) compared to Çelik et al. (11 (18.0%)) may be attributed to referral bias in tertiary care settings [18].

Early fixation was achieved in 17 (85.0%) patients within 24 hours, consistent with Anjum et al., who reported early stabilization in all cases [19]. The mean hospital stay of 8.3 ± 1.0 days was comparable to previous studies, reflecting the benefits of early stabilization and mobilization [12,19]. Bone loss was observed in seven (35.0%) patients, with corticotomy performed in six (30.0%), highlighting the advantage of the Ilizarov technique in managing bone defects. Madhusudhan et al. demonstrated similar effectiveness of bone transport in managing defects up to 11 cm [21].

Pin tract infection was the most common complication in the present study, observed in 11 (55.0%) patients, predominantly Grade II (8 (40.0%)), which is comparable to Bhosale and Naikwade (11 (36.67%)) but higher than Ali et al. (5 (25.0%)) [20,22]. Importantly, no deep infections or non-unions were observed, which is consistent with Ali et al. [22]. Minor deformities such as limb length discrepancy (0.49 ± 0.32 cm) and angulation were within acceptable limits, similar to findings reported by Çelik et al. and Bhosale and Naikwade, suggesting effective maintenance of alignment with circular fixation [18,20].

All patients achieved union (20 (100.0%)) with a mean union time of 24.1 ± 3.4 weeks, comparable to Bhadra et al. (24.0 weeks) and Ali et al. (20.76 weeks), and superior to Thomas and Nishara, who reported a union rate of 41 (74.5%) [12,22,23]. Functional outcomes were favourable, with excellent results in 15 (75.0%) patients and good outcomes in 3 (15.0%), comparable to Bhosale and Naikwade (24 (80.0%)) excellent [20]. Early weight bearing was achieved in all patients (20 (100.0%)) by day 2, similar to Sidharthan et al., emphasizing the advantage of Ilizarov fixation in promoting early mobilization and functional recovery [24]. A randomized controlled trial by Sharma et al. demonstrated comparable union rates between hybrid Ilizarov fixation and distal tibial locking plate fixation, with the Ilizarov method offering advantages in early weight bearing and better management of soft tissue conditions [25]. This supports the findings of the present study, particularly in the context of complex open fractures where preservation of soft tissue biology is crucial.

The present study showed no significant association between mode of injury or Gustilo-Anderson grade and final outcome (p > 0.05), indicating consistent results across different injury severities. This differs from Çelik et al., who reported worse outcomes with increasing fracture severity, possibly due to inclusion of grade IIIC fractures in their cohort [18]. Comparative studies such as Bhadra et al. have also demonstrated superior outcomes of Ilizarov fixation over conventional external fixators in terms of union rate, alignment, and complication profile, supporting its role as an effective modality in the management of complex open tibial fractures [23]. The favourable outcomes observed in the present study may also be attributed to the biomechanical stability and biological advantages of the Ilizarov technique, including enhanced osteogenesis, preservation of periosteal blood supply, and facilitation of soft tissue healing through the principle of tension-stress.

Strengths and limitations

The present study has several strengths, including its prospective design, uniform treatment protocol using the Ilizarov technique, and comprehensive assessment of clinical, radiological, and functional outcomes using standardized criteria. The use of a fracture-specific functional scoring system further enhances the comparability of results with existing Ilizarov-based literature. The inclusion of only Gustilo-Anderson grades II, IIIA, and IIIB fractures allowed focused evaluation of moderate to severe open injuries. However, the study has certain limitations, including a relatively small sample size (n = 20), absence of a control or comparative group, and single-centre design, which may limit generalizability. Additionally, although follow-up was adequate to assess union and early functional outcomes, longer follow-up is required to evaluate late complications such as post-traumatic arthritis or long-term functional deficits.

## Conclusions

The Ilizarov external fixator appears to be an effective and reliable modality for the management of open tibial fractures, offering stable fixation, early mobilization, and satisfactory functional recovery with an acceptable complication profile. Its versatility in managing complex fracture patterns, including those with soft tissue compromise and bone loss, makes it a valuable option in orthopaedic practice. The high union rate, early weight bearing, and favourable functional outcomes observed in the present study further support its clinical utility. However, these results should be interpreted with caution in view of the study limitations. Further large-scale, multicentric, randomized controlled trials with longer follow-up are needed to validate these observations, compare outcomes with other treatment modalities, and incorporate standardized patient-reported outcome measures for comprehensive assessment.
